# Bone Turnover in Azerbaijani Patients with Type 2 Diabetes

**DOI:** 10.18502/ijph.v49i10.4711

**Published:** 2020-10

**Authors:** Sain Sattar SAFAROVA

**Affiliations:** Department of Internal Medicine, Azerbaijan Medical University, Baku, Azerbaijan

## Dear Editor-in-Chief

A few studies linked changes of bone turnover in the context of type 1 diabetes complications, while numerous studies do not addressed bone disorders to type 2 diabetes (T2DM) complications (1).

Our cross-sectional study was conducted among 137 patients (female: 85 / male: 52) patients in Baku, Azerbaijan with T2DM. We aimed to assess some morphological and functional properties of bone tissue to suggest recommend screening of osteoporosis in that group of patients.

We observed some trends between changes in BMD and bone markers and their association with other metabolic parameters in T2DM. The association between procollagen type 1 N-terminal propeptide (P1NP) and C-terminal cross-linking telopeptide of type I collagen (b-CTX) with kidney function was revealed. This indicated that impairment of kidney function in patients with diabetes leads to a decrease in renal clearance of bone markers, which may at least partially explain the increase in the diagnostic significance of the latter in patients with T2DM. These data are also confirmed in the literature (1,2)

The negative association of P1NP with glycated haemoglobin (HbA1c) indicates that the formation of bone tissue is disturbed by high blood glucose levels; accordingly, people with T2DM and poor glycemic control are at greater risk of fragility fractures (3), although the mechanisms are not clear until the end. P1NP levels did not show a statistically significant decrease, which indicates a fairly high metabolic activity, against the background of a failed attempt by osteoblasts to compensate for increased bone resorption. Because P1NP indicates osteoblastic function in patients with diabetes, bone formation is not effective enough to compensate bone resorption.

These results are consistent with findings of other authors, who have studied bone mineral density (BMD) of the axial and appendicular skeleton in T2DM (4). Patients with T2DM had a higher level of CTx, a marker of bone destruction, which reflects a decrease in bone metabolism compared to control, regardless of age and duration of the disease. Evaluation of alkaline phosphatase and osteocalcin was within the norm and was a small informative method to evaluate bone turnover, confirmed by the data of other authors (5).Also, according to the results of data analysis, it can be argued that there is a positive relationship between body mass index (BMI) and bone mineral density, which is confirmed by the data of other authors (3). However, further study and confirmation of the negative effect of disease duration on the processes occurring in bone tissue in diabetes are needed.

Most studies that have investigated the relationship between GFR and bone metabolism, relying on creatinine values as an integrative measure of renal function, but changes in blood albumin concentration are also associated with risk factors for osteoporosis, since albumin regulates calcium transport to bone. Hypoalbuminemia could exert directly and indirect effect on bone turnover which leads to shift the balance towards to resorption processes by acting on the nuclear factor-kappa B, inflammatory processes and antioxidant activities that reduced bone mineral density, resulting in a decrease of calcium phosphate (hydroxyapatite) crystals formation, and also affects concentration of parathyroid hormone (PTH) and vitamin D3 (2).

In conditions of decreased renal function, the synthesis of renal 1-α hydroxylase, which transfers vitamin D3 into the active form, is reduced, which reduces intestinal calcium absorption and activates the production of PTH by the parathyroid gland, resulting in the elution of calcium and phosphorus from the bones. On the increase of the latter reacts fibroblast growth factor-23 (FGF23), the increase of which is much faster and easier to remove phosphate in the urine and inhibits the activation of vitamin D, resulting in a decrease in the content of calcium in the blood and then in the tissues (5). Obviously, the relative insulin secretion deficiency leads to hypersecretion of PTH associated with decreased levels of serum Ca and causes secondary hyperparathyroidism, promoting bone decalcification, which is consistent with previous study (6). However, despite the clinical availability of alkaline phosphatase, its use as a bone marker is poorly informative, due to its synthesis in various tissues.

Consequently, changes in the functions of these organs affect its concentration, and therefore it cannot be asserted that this biochemical indicator of bone remodeling is specific for the diagnosis of diabetic osteopathy. We agree with other studies (4, 6) where results demonstrated that bone remodeling markers levels indicate increased bone resorption. The results of densitometry indicate the absence of significant differences in BMD between the patients with T2DM and control. Between the duration of T2DM and decrease in BMD there is a relationship, most expressed in men under the age of 50 yr.
